# Synergism in hyperhomocysteinemia and diabetes: role of PPAR gamma and tempol

**DOI:** 10.1186/1475-2840-9-49

**Published:** 2010-09-09

**Authors:** Paras K Mishra, Neetu Tyagi, Utpal Sen, Irving G Joshua, Suresh C Tyagi

**Affiliations:** 1 Department of Physiology & Biophysics, School of Medicine, University of Louisville, Louisville, Kentucky-40202, USA

## Abstract

**Background:**

Hyperhomocysteinemia (HHcy) and hyperglycemia cause diabetic cardiomyopathy by inducing oxidative stress and attenuating peroxisome proliferator- activated receptor (PPAR) gamma. However, their synergistic contribution is not clear.

**Methods:**

Diabetic Akita (Ins2+/-) and hyperhomocysteinemic cystathionine beta synthase mutant (CBS+/-) were used for M-mode echocardiography at the age of four and twenty four weeks. The cardiac rings from WT, Akita and hybrid (Ins2+/-/CBS+/-) of Akita and CBS+/- were treated with different doses of acetylcholine (an endothelial dependent vasodilator). High performance liquid chromatography (HPLC) was performed for determining plasma homocysteine (Hcy) level in the above groups. Akita was treated with ciglitazone (CZ) - a PPAR gamma agonist and tempol-an anti-oxidant, separately and their effects on cardiac remodeling were assessed.

**Results:**

At twenty four week, Akita mice were hyperglycemic and HHcy. They have increased end diastolic diameter (EDD). In their heart PPAR gamma, tissue inhibitor of metalloproteinase-4 (TIMP-4) and anti-oxidant thioredoxin were attenuated whereas matrix metalloproteinase (MMP)-9, TIMP-3 and NADPH oxidase 4 (NOX4) were induced. Interestingly, they showed synergism between HHcy and hyperglycemia for endothelial-myocyte (E-M) uncoupling. Additionally, treatment with CZ alleviated MMP-9 activity and fibrosis, and improved EDD. On the other hand, treatment with tempol reversed cardiac remodeling in part by restoring the expressions of TIMP-3,-4, thioredoxin and MMP-9.

**Conclusions:**

Endogenous homocysteine exacerbates diabetic cardiomyopathy by attenuating PPAR gamma and inducing E-M uncoupling leading to diastolic dysfunction. PPAR gamma agonist and tempol mitigates oxidative stress and ameliorates diastolic dysfunction in diabetes.

## Background

Hyperhomocysteinemia (HHcy) is an independent cause of cardiovascular diseases [1,2]. In diabetes, plasma homocysteine level is elevated [3,4]. Both hyperglycemia and HHcy lead to diabetic cardiomyopathy, which is a leading cause of morbidity and mortality [5-9]. A positive correlation exits between plasma homocysteine (Hcy) and left ventricular hypertrophy (LVH) in diabetes [10]. A direct link between Hcy and cardiovascular diseases is evident from the fact that every 3 μM/L increase in Hcy level contributes to 10% increase in the risk of coronary heart disease and 20% more chances of stroke [11]. Hcy induces cardiac hypertrophy in rats [12,13] and instigates bradycardia [14,15]. Hcy engenders oxidative stress by generating reactive oxygen species (ROS) [16,17]. In diabetes, Hcy mediated generation of oxidative radicals induce matrix metalloproteinases (MMPs) and inhibit their tissue inhibitors (TIMPs) that result into extracellular matrix (ECM) remodeling [18,19]. Additionally, activation of MMPs degrades endothelial basement membrane [20] that connects endothelium to myocytes through ECM and adhesion molecules. The disruption of connection between endothelium (E) and myocytes (M) causes E-M uncoupling [21,22] resulting into defective diastolic relaxation. There are evidences suggesting the effect of Hcy on hypertension [23], diabetes [4] and insulin resistance [24]. However, synergism between Hcy and hyperglycemia in diabetic cardiomyopathy is unclear.

The insulin2 mutant (Ins2+/-) Akita has been used as a genetic model for type1 diabetes. Due to heterozygous mutation in Insulin2 gene (located on chromosome 7), the proinsulin2 is misfolded and cannot exocytose from pancreatic beta cells resulting into accumulation and ultimately death of beta cells. These mice are normoglycemic until the age of three week and blood glucose level shoots up constantly after three-four weeks reaching robust hyperglycemia at ten week. Akita has been used as a model system for diabetes [25]. On the other hand, cystathionine beta synthase mutant (CBS+/-) is widely used as a model system for HHcy. CBS gene is located on chromosome 17 and is required for metabolism of Hcy by transsulfuration pathway [26-29]. The double knock out of Akita and CBS+/- is not available. To investigate individual and synergistic effect of hyperglycemia and HHcy, we cross-bred Akita with CBS+/- to obtain four types of offspring: Ins2+/+/CBS+/+ (WT); Ins2+/-, CBS+/+ (Akita); Ins2+/+/CBS+/- (CBS mutant) and Ins2+/-/CBS+/- (double knock out).

Glucose mediated oxidative stress is mitigated by peroxisome proliferator- activated receptors (PPARs). They are ligand-activated transcription factors that play pivotal role in regulation of genes involved in hyperglycemia, lipid metabolism, vascular tone and inflammation [30-32]. PPARγ contributes in ameliorating diabetic complications [3,33,34]. The PPARγ agonists modulate insulin resistant [33,34] and activated PPARγ inhibits MMP activation [35]. Interestingly, PPARγ cannot decrease plasma Hcy [18,36-38]. Nevertheless, it is attenuated by HHcy [39]. In a ventricular pressure overload model of mice, reduced expression of PPARγ causes left ventricular hypertrophy [40]. The PPARγ agonist (PGJ2) showed a competition for binding to PPARγ with Hcy [41] and ameliorates diabetic cardiac hypertrophy [42]. However, the independent and synergistic effect of Hcy and PPARγ in cardiac hypertrophy and matrix remodeling in diabetes is nebulous.

HHcy engenders oxidative stress by inducing reactive oxygen species (ROS) and enhances NADPH oxidase 4 (NOX4) [43]. ROS is attenuated by tempol, an anti-oxidant [43,44]. The use of anti-oxidant to mitigate the effect of oxidative stress is a promising approach for treating diabetic cardiomyopathy [45]. ROS also affects nitric oxide (NO) that in contact with superoxide generates highly reactive peroxynitrite (ONOO^-^), which induces cascade of pathological signaling. Nitric oxide (NO) is a vasodilator and is considered as cardioprotective molecule that maintains E-M coupling. This study was designed to investigate association between Hcy and glucose in structural and functional remodeling of endocardium by defining their link to PPARγ and NO metabolism in diabetes. We hypothesize that E-M uncoupling in diabetes is due in part to increased level of homocysteine causing activation of latent MMP-9, attenuation of thioredoxin and TIMP-4 in response to antagonizing PPARγ.

## Methods

### Animal model

Akita, CBS+/- and C57 BL/6J (WT) were procured from Jackson Laboratory (Bar Harbor, ME). To avoid gender specific complexity, only male mice were used for the experiments. They were housed in the animal care facility of University of Louisville and fed standard mouse chow diet. The animal room was maintained at 22-24°C with 12:12 hour light-dark cycle. At the end of experiments, animals were sacrificed following the protocol approved by Institutional Animal Care and Use Committee of University of Louisville. Further, animal care and use programs were carried out according to standard protocol and guidelines of National Institute of Health (NIH) and *Guide for the Care and Use of Laboratory Animals *(NIH Pub. No. 86-23, revised 1985) and regulation of Animal Welfare Act.

### Genotyping of Akita and CBS mutant mice

DNA was extracted from the tail tip of mice at the age of six to eight weeks and was amplified by PCR using specific primer sequences. The primer sequence for Ins2 gene was designed from the region of exon 3 because Ins2 mutation in Akita disrupts *Fnu 4 H*I site in the exon 3 of Ins2 and digestion with *Fnu 4 H*I did not change the size of PCR product from mutated allele (280bp), rather it decreased the size of the wild type allele to 140 bp. The primer sequences were forward: 5'TGC TGAT GCC CTG GCC TGCT 3'; reverse: 5' CAC ATA TGC ACA TG 3'. For CBS heterozygous mutation, primer sequences were designed from intron 3 flanking the region of neo insert in CBS gene [46]. The primer sequences were forward: 5' GCCTCTGTCTGCTAACCTA3'; reverse: 5' GAGGTCGACGG TATCGATA3'. The PCR product of CBS from wild type is 800 bp, while the disrupted/mutant allele was 180 bp.

### Double knock out and treatment groups

To obtain the double knock out of Akita and CBS+/- (Ins2+/-/CBS+/-), female Akita were crossed with male CBS+/- (2♀: 1♂). Four types of offspring were produced: WT, Akita, CBS+/- and double knock out (Ins2+/-/CBS+/-). At the age of twenty week, these offspring were treated with vehicle (normal drinking water), PPARγ agonist ciglitazone (CZ-Cal Biochem Corp, CA, USA) -8 μg/ml and anti-oxidant tempol (Sigma Aldrich, USA)-10 mM/L for four weeks through drinking water. Both CZ and PPARγ bind at the micromolar range [31]. Assuming the total blood volume 2 ml and daily drinking water intake 5 ml, the 8 μg/ml of CZ in drinking water added into the blood ~ 2 mg CZ/kg body weight/day producing a blood concentration of ~32 μM/L, which was enough to saturate the binding sites of PPARγ [47]. To rule out the other effects of PPARγ agonist [48], food and water intake was measured every second day during the treatment. Additionally, liver was weighed at the end of experiments (data not shown) to confirm that there was no peroxisome proliferation and hepatotoxicity [49] after treatment with CZ as reported with other PPARγ-agonists [50].

### Blood glucose measurement

A glucometer with one-touch strip was used to measure the blood glucose level in mg/dL.

### HPLC for Hcy level measurement

Blood drawn from the dorsal aorta of mice with the help of heparin rinsed syringe was collected in an Eppendorf tube on ice. It was immediately centrifuged at the rate of 5000 rpm for 10 min at 4°C. The clear plasma was collected and stored at -80°C and used for HPLC. Hcy in the plasma samples was detected using a Shimadzu Class-VP 5.0 chromatograph (Shimadzu) as described previously [51].

### Echocardiography

M-mode echocardiogram was obtained from a SONO-5500 echocardiographic system equipped with a 12 -MHz phased- array transducer. The transducer was placed on the left hemithorax of mice so that echocardiogram could be taken from a short-axis view of the left ventricle at or just below the tip of the mitral-valve leaflet. Only those echocardiograms, which showed well-defined continuous interfaces of the septum and posterior wall, were collected and ventricle volume and axis lengths were determined.

### Cardiac ring preparation and study of relaxation response

Transverse sections of approximately 2-3 mm thickness that appeared as "doughnut" was prepared from left ventricle (LV) after removing the right ventricle. The heart was sectioned transmurally in such a manner that LV rings (cardiac ring) of even thickness could be obtained. The rings were immediately mounted on a polygraph in a tissue myobath containing physiological saline solution (PSS-118 mM NaCl, 4.7 mM KCl, 2.5 mM CaCl_2_, 1.2 KH_2_PO_4_, 1.2 MgSO_4_, and 11.2 Dextrose) maintained at 37°C. After stretching the ring, it was brought to the resting tension where 20 mM CaCl_2 _was applied. When the contraction was maximum, different doses of acetylcholine was added to myobath. Acetylcholine stimulates endothelial dependent contraction. To avoid the orientation dependent variation in contraction measurement, two measurements at the rotation of cardiac rings at 90° was determined and average was used. The maximum contraction by 20 mM CaCl_2 _was taken as 100% contraction and percentage relaxation was calculated as a fraction of 100% contraction. Further, to avoid ischemia, the myobath was bubbled with a mixture of 95% oxygen and 5% carbon dioxide throughout the experiment and the experiment was completed within 40 min [52].

### RT-PCR

The reverse-transcription polymerase chain reaction was performed after extracting RNA from left ventricle. The Promega RT-kit was used for reverse transcription following the method as described elsewhere [53]. The mRNA levels of PPARγ, NOX4, Trx, MMP-2, -9 and GAPDH were determined by RT-PCR using following primers: Thioredoxin (Trx) sense: 5' TGGATCCATTTCCATCTGGT 3' antisense: 5' CCTTGTTAGCACCGGAGAAC 3', GAPDH sense: 5' TGAAGGTCG GTGTG AAC GGATTTGGC 3' antisense: 5' CATGTAGGCCATGAGGTCCACCAC3', MMP-2 sense: 5' GCACTCTGGAGCGAGGATAC 3' antisense: 5'GCCCTCCTAAGCCAGTCTCT3', MMP -9 sense: 5' AAGGCAAACCCTGTGTGTTC3' antisense: 5'GTGGTTCAGTT GTGGTGGTG3', NOX4 sense: 5' CCA GAA TGA GGA TCC CAG AA 3' antisense: 5' TGG AAC TTG GGT TCT TCC AG3', PPARγ sense: 5' ATGGCCATTGAGTGCCGAGTCTG3' antisense: 5'GGCTTTTGAGGAACTCCCTGGT CA3'. The PCR program for MMP-2,-9 and GAPDH was 94°C-2 min, [94°C -30 sec, 57°C -30 sec, 72°C -1 min] x30, 72°C-2 min. For Trx and Nox4, the PCR programs were 94°C-5 min, [94°C-40 sec, 55°C-40 sec, 72°C-1 min] × 30, 72°C-5 min and 94°C-2 min, [94°C-1 min, 53°C- 1 min, 72°C-1 min] × 30, 72°C -5 min, respectively.

### Protein extraction and Western Blotting

The left ventricle from freshly extracted heart was homogenized in Ripa buffer (Boston BioProducts, Worcester, MA, USA) with proteinase inhibitor cocktail (Sigma, Saint Louis, MO, USA) and centrifuged at 5000 rpm for 5 min at 4°C. The supernatant was collected. The concentration of proteins was estimated by Bradford method as described elsewhere [53]. The Western blotting was performed by following the same protocol as described earlier [53].

### Statistical analyses

At least five mice were used from each group and data were presented as mean and standard deviation (SD). Student t-test was used and a value of p < 0.05 was considered as significant.

## Results

**Figure 1:**
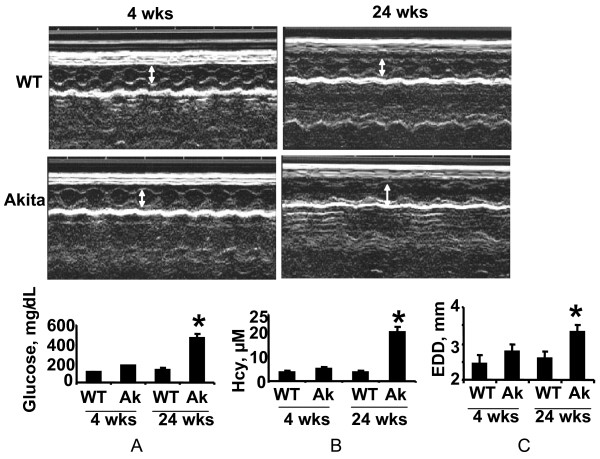
**A: Measurement and comparison of blood glucose level: Blood glucose level was determined at four and twenty four weeks in WT and Ins2+/- that showed robust hyperglycemia at twenty four week Akita**. **B: **Determination and comparison of plasma homocysteine (Hcy) level: Plasma Hcy level was determined by high performance liquid chromatography (HPLC) at four and twenty four weeks in male WT and Ins2+/-. There was significant elevation in Hcy level at twenty four week in Akita. **C: **Echocardiogram and end diastolic diameter (EDD): Echocardiography was performed at four and twenty four weeks in male WT and Ins2+/- using SONO 5500 device. The diastolic diameter is shown by double headed arrow. It was increased at twenty four week in Akita. The end diastolic diameter (EDD) was significantly increased at twenty four week in Akita. Each bar represents average +SD of ten animals. *, p < 0.05 compared to WT.

**Figure 2:**
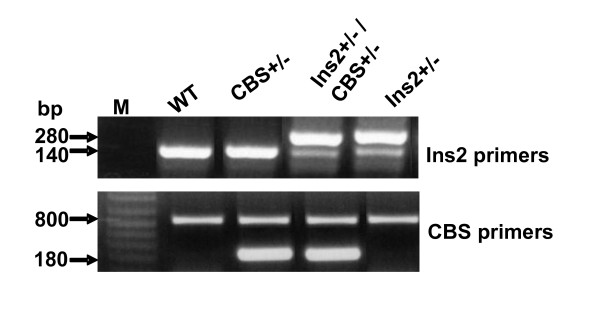
**Genotyping of Ins2+/-, CBS +/- and Ins2+/-/CBS+/-**. Two PCR products suggest the heterozygous mutation, while single band represents WT allele. In Ins2+/- and CBS +/-, the double bands of specific PCR product confirmed the heterozygous mutation of Ins2 and CBS genes, respectively.

**Figure 3:**
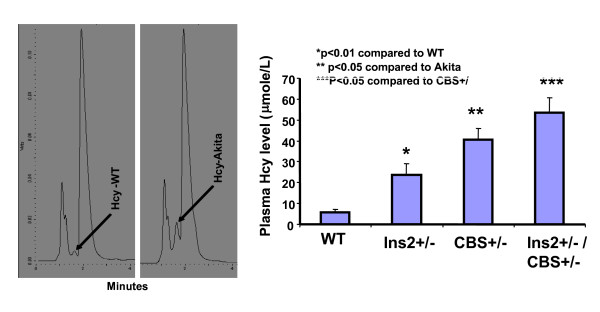
**Determination and comparison of plasma Hcy level by HPLC: The peak of plasma Hcy was shown in WT and Ins2+/-**. Hcy level was determined and compared among WT, Ins2+/-, CBS +/- and Ins2+/-/CBS+/-. Each bar represents average +SD from six animals. *, p < 0.05 compared to WT; ** p < 0.05 compared to Ins2+/-, and ***, p < 0.05 compared to CBS+/-.

**Figure 4:**
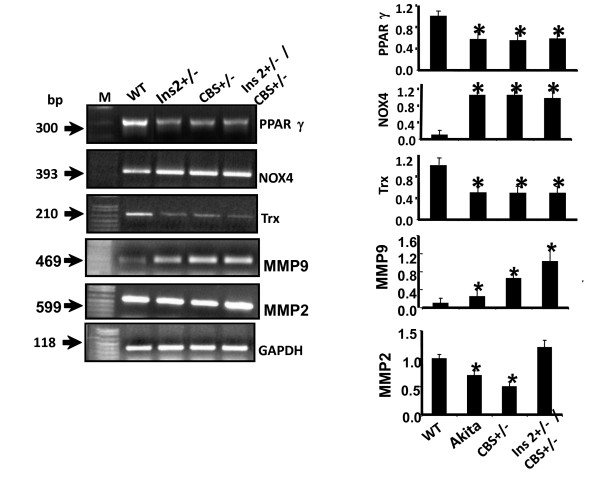
**Expression of PPARγ, oxidative radicals and MMPs: RT-PCR of NOX -4, PPARγ, thioredoxin (Trx) and MMP-2,-9 from the heart tissue of Ins2+/-, CBS +/- and Ins2+/-/CBS+/-**. GAPDH was used as a loading control. The bar graph is made from the scanned arbitrary unit showing mean and standard deviation of eight animals. *, p < 0.05.

**Figure 5:**
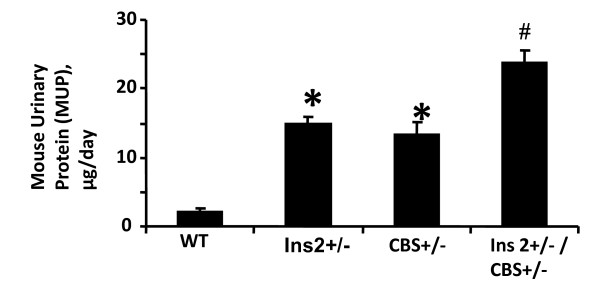
**The protein content in urine was estimated on daily basis for 7 days and mean urinary proteins is shown with standard deviation for WT, Ins2+/-, CBS+/- and Ins2+/-/CBS+/-**. n = 5; *, p < 0.05 compared to WT; #, p < 05 compared to CBS+/-.

**Figure 6:**
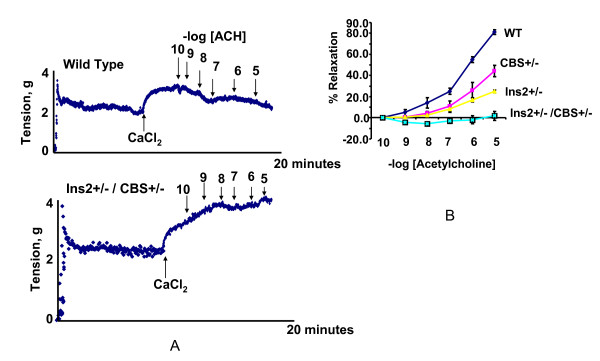
**Effect of acetylcholine (ACH) on contraction of cardiac muscle**. Calcium chloride was applied on the cardiac ring (uniform transverse section of the LV) for maximum pre-contraction followed by dose dependant treatment of ACH. **A**. The upper and lower panels represent the contraction pattern of WT and Ins2+/-/CBS+/-, respectively. **B**. The percentage relaxation (indicating the potential of contraction) was calculated with respect to the maximum contraction after treatment with 20 mM CaCl_2 _and compared among WT, CBS+/-, Ins2+/- and Ins2+/-/CBS+/-. The capability of contraction decreased in the increasing order of CBS +/-, Ins2+/- and Ins2+/-/CBS+/- when compared to WT. Each data point represents mean ± SD of at least six animals.

The comparison of blood glucose level of Akita and WT at four and twenty four weeks revealed that at four week both WT and Akita are normoglycemic. However, there was significant increase in the blood glucose level at twenty four week in Akita (Figure 1A). To investigate the effect of hyperglycemia on Hcy, plasma Hcy level was determined in the same groups of animals. Concurrent with the glucose level, there was significant elevation in Hcy level in twenty four week Akita (Figure 1B). To determine whether elevation of glucose and Hcy level had effect on the cardiac function, M-mode echocardiography was performed, which showed increase in end diastolic diameter (EDD) in twenty four week Akita (Figure 1C). Since both glucose and Hcy level was enhanced in Akita (Figure 1A, B), the independent and/or synergistic effect of Hcy and glucose on cardiac dysfunction was determined by using double knock out (Ins2+/-/CBS+/-). The genotypes of the mutants were confirmed by PCR using Ins2 and CBS specific primers (Figure 2). The double bands represent heterozygous mutation (Figure 2). The plasma Hcy level was determined in the mutants and compared with WT. Hcy level was significantly high in both CBS+/- and Ins2+/- when compared with WT (Figure 3). Interestingly, in double knock out, Hcy level increased almost two-fold as compared to that of Akita. Notably, the elevation of Hcy level was significantly (p < 0.05) higher in double knock out when compared to CBS+/- (Figure 3). The expression of PPARγ, NOX-4 and Trx was measured in all four groups (WT, Ins2+/-, CBS+/- and CBS+/-/Ins2+/-). It revealed a significant increase in NOX-4, while decrease in PPARγ and Trx in all the three mutants (CBS+/-, Ins2+/- and Ins2+/-/CBS+/-) (Figure 4). Further, there was significant up regulation of MMP-9 and down regulation of MMP-2 in CBS+/- (Figure 4). Remarkably, the total urinary protein concentration was increased in the above three mutants (Figure 5). To determine the E-M uncoupling, cardiac rings from the four groups were treated with different doses of acetylcholine after pre-constriction by CaCl_2 _and their rate of relaxation was measured (Figure 6A). There was a clear gradient decrease in the rate of relaxation in WT, CBS+/-, Ins2+/- and Ins2+/-/CBS+/- (Figure 6B).

**Figure 7:**
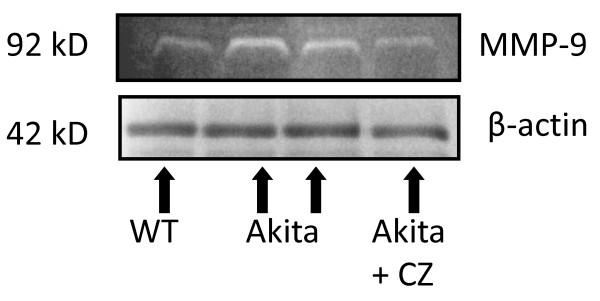
**In - gel gelatin Zymography to determine the activity of MMP-9 in WT, Ins2+/-and Ins2+/- treated with ciglitazone**. Beta-actin was used as a loading control. The collagen substrate chelating represents the activity of MMP-9 and indicated by white bands. It was higher in Akita (two bands in the middle) as compared to WT. The treatment with ciglitazone reduced the activity of MMP-9 in Akita.

**Figure 8:**
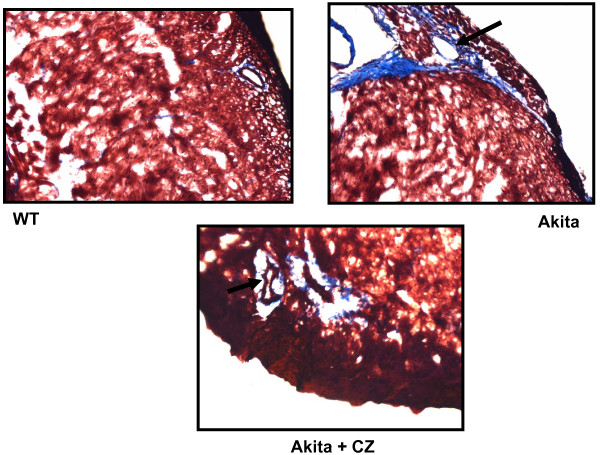
**The Masson Trichrome staining of histological sections of the heart from WT, Ins2+/- and Ins2+/- treated with ciglitazone**. The blue stain represents collagen deposition. Collagen deposition was increased in Ins2+/- and attenuated after treatment with ciglitazone.

**Figure 9:**
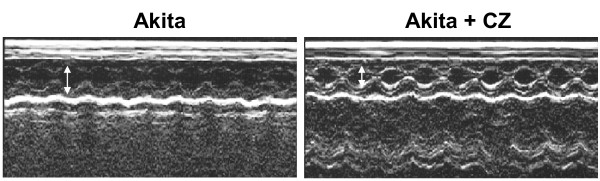
**Diastolic diameter of Ins2+/- with and without treatment with ciglitazone**. The echocardiogram shows amelioration of diastolic diameter (double headed arrow) of Ins2+/- after treatment with ciglitazone.

**Figure 10:**
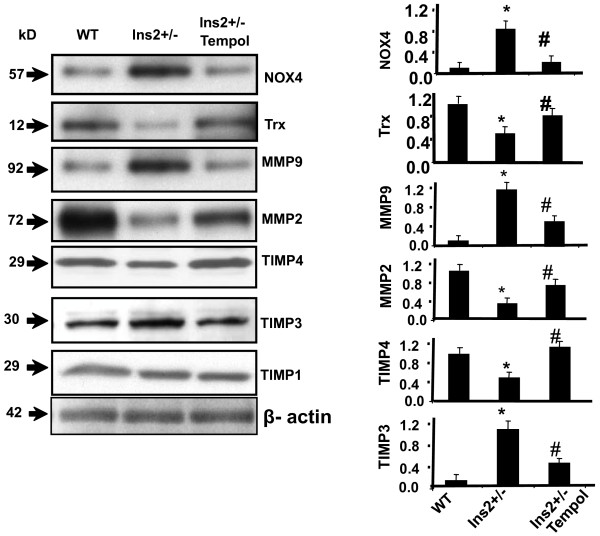
**Effect of tempol (anti-oxidant) on Ins2+/-: Western blots showing expression of NOX-4, thioredoxin (Trx), MMP-2,-9 and TIMP-1,-3,-4 in the heart tissue of WT, Ins2+/- and Ins2+/- treated with tempol (Akita/Tempol)**. (i) The expression of NOX-4 increased and Trx decreased in Akita as compared to WT. However, it was attenuated after treatment with tempol. The expression of MMP-9 and TIMP-3 was increased, while MMP-2 and TIMP-4 was decreased in Akita. The treatment with tempol ameliorated the expression of MMP-2,-9 and TIMP-3,-4. There was no change in the expression of TIMP-1. Beta-actin was used as a loading control. (ii) Bar graph was based on the scanned arbitrary unit of the bands and represents the mean ± SD of six animals. *, p < 0.05 to WT; #, p < 0.05 to Akita.

To determine the role of PPARγ in diabetic cardiomyopathy, Akita was treated with CZ. The activity of MMP-9 was determined in WT and Akita with and without treatment with CZ. There was up regulation of MMP-9 activity in Akita. However, it was attenuated after treatment with CZ (Figure 7). The collagen deposition, which indicates fibrosis, was also decreased in Akita after treatment with CZ (Figure 8). The diastolic function is reflected through diastolic diameter. It was improved in CZ treated Akita (Figure 9). To determine the effect of oxidative stress on diabetic cardiomyopathy, Akita was treated with tempol, an anti-oxidant. The results showed mitigation of NOX-4 and enhancement of Trx level in tempol treated Akita (Figure 10). Additionally, MMP-9 and TIMP-3 were attenuated, while MMP-2 and TIMP-4 were induced in tempol treated Akita (Figure 10). Nevertheless, there was no change in the TIMP-1 expression (Figure 10).

## Discussion

There are two insulin genes in mice: insulin 1 and insulin 2. Insulin 2 is predominantly involved in glucose metabolism and is homologous to human insulin. Recent clinical data revealed that mutation in insulin 2 is a major cause of neonatal diabetes [54]. Therefore, insulin 2 mutant Akita mouse that resembles human hypoinsulinemia has been used for investigating the mechanism of diabetic cardiomyopathy. Hypoinsulinemia in later stage leads to insulin insensitivity- a feature of type 2 diabetes [55]. The four groups obtained by cross- breeding Ins2+/- with CBS+/- provides insight into the independent and synergistic implications of HHcy and hyperglycemia on diabetic cardiomyopathy. The proinsulin maturation and exportation to cytoplasm occurs after the developmental age of three to four weeks. Therefore, glucose and Hcy level was determined and compared between WT and Akita at four week. We did not find any significant change in glucose and Hcy level and end diastolic diameter (EDD) at four week in Akita. However, there was robust hyperglycemia and HHcy accompanied by increase in EDD at twenty four week (Figure 1A, B, C). The increase in EDD at twenty four week (Figure 1C) suggests diastolic dysfunction. These results support previous findings of diabetic cardiomyopathy in Akita [24,25,54,55].

Hybrids of Ins2+/- and CBS+/- were confirmed by genotyping (Figure 2). The two-fold increase in glucose and Hcy level in Ins2+/-/CBS+/- suggests synergism in HHcy and hyperglycemia. This finding tempted us to speculate that Ins2 gene might be interfering with Hcy metabolism and inducing hyperhomocysteinemia in CBS+/- (Figure 3). However, further investigations are required to dissect the detailed mechanism. Oxidative stress generated by HHcy [53] is mitigated by PPARγ. The analyses of results (Figure 4) revealed increase in oxidative stress in Ins2+/-, CBS+/- and Ins2+/-/CBS+/- as compared to WT due in part to attenuation of PPARγ [41,42]. The increase in oxidative stress in HHcy and diabetic mice extend support to the other published reports [16-18,43]. The down regulation of PPARγ in diabetic mice is consistent with the previous finding [21].

Increase in oxidative radicals activates latent MMPs. It is found that MMP-2 and MMP-9 play important role in cardiac remodeling [56-59]. Therefore, we determined the individual and combined effect of hyperglycemia and HHcy on MMP-2 and MMP-9. The results revealed induction of MMP-9 both in Akita and CBS+/-. Interestingly, there was synergism between hyperglycemia and HHcy for MMP-9 induction (Figure 4). MMP-2 was attenuated both in Akita and CBS+/- but remained unchanged in Ins2+/-/CBS+/- (Figure 4). It indicates that the active site for inducing MMP-2 for either Akita or CBS+/- was antagonized or inhibited in Ins2+/-/CBS+/-. The induction of MMP-9 in HHcy (CBS+/-) and hyperglycemia (Ins2+/-) due to oxidative stress is in line with the earlier findings [17,20,53,56].

Chronic diabetes ultimately leads to multi-organ failure. The alteration in the amount of urinary protein indicates the internal damage of the body [60]. The total urinary protein of twenty four week Akita, CBS+/- and Ins2+/-/CBS+/- was compared with WT (Figure 5). We found an increased level of total urinary protein in both Akita and CBS+/-. Interestingly, the total urinary protein was almost two fold increased in Ins2+/-/CBS+/- suggesting synergistic effect of hyperglycemia and HHcy (Figure 5).

Extracellular matrix bridges endothelium and myocyte, which is essential for maintaining the E-M coupling. The E-M coupling is required for the synchronized beating of cardiomyocytes, and proper systolic contraction and diastolic relaxation of the heart. Defective E-M coupling leads to heart failure. For proper contraction of cardiomyocytes, calcium ion is indispensable. The cardiac rings from the four groups were exposed to 20 mM CaCl_2 _that allows maximum contraction as shown in the representative graph (Figure 6A). After maximum contraction, cardiac rings were exposed to increasing doses of acetylcholine that allows endothelial dependent contraction. The percentage relaxation was recorded from the maximum contraction (Figure 6B). The higher percentage relaxation suggests healthy heart as shown by WT (Figure 6B). We found decrease in the percentage relaxation in CBS+/- and Akita (Figure 6B). In Ins2+/-/CBS+/-, there was almost no capacity for relaxation suggesting deterioration of E-M coupling (Figure 6B). These findings reinforce synergism between hyperglycemia and HHcy for E-M uncoupling in Akita. It supports the previous finding that E-M uncoupling is one of the major causes of diabetic cardiomyopathy [21].

To confirm the role of PPARγ in diabetic cardiomyopathy, Akita was treated with CZ- a PPARγ agonist. Since MMP-9 is elevated in Akita (Figure 4) and it causes fibrosis [58], the activity of MMP-9 and level of fibrosis was determined in Akita after treatment with CZ and compared with WT. In twenty four week Akita, the activity of MMP-9 (Figure 7) and fibrosis (Figure 8) was induced. However, Akita treated with CZ showed reduced MMP-9 activity and less fibrosis as compared to the untreated group (Figure 7, 8). As PPARγ inhibits the oxidative radicals, it was assumed that the activity of MMP-9 would be inhibited and thereby fibrosis would be ameliorated. The therapeutic effect of CZ was also confirmed by echocardiography, where EDD was decreased in CZ treated Akita (Figure 9). These findings suggest that PPARγ mitigates the effect of MMP-9 and has cardio-protective role in diabetes. It is consistent with the finding that activation of PPARγ improves endothelial function and thereby mitigates the diabetic cardiomyopathy [30]. Further, PPARγ is also implicated in the modulation of metabolic syndrome that causes cardiovascular complications [30]. The critical role of PPARγ in diabetes and cardiovascular diseases suggest that it can be used as a therapeutic target for diabetic cardiomyopathy.

To investigate the generation of oxidative radicals, the levels of oxidant (NOX-4) and anti-oxidant (Trx) was determined in WT and Akita. The up regulation of NOX-4 and down regulation of Trx suggests oxidative stress in Akita (Figure 10). TIMP-1 is associated with fibrosis [58,61,62], while TIMP-3 [58,63] is associated with apoptosis. Contrary to our expectation, there was no significant change in the expression of TIMP-1 (Figure 10) suggesting that anti-oxidant tempol does not have direct effect on TIMP-1. However, TIMP-3 was inhibited by tempol (Figure 10) indicating that tempol mitigates apoptosis by inhibiting oxidative radicals. TIMP-4 is abundant in the heart and has cardio-protective role [58]. Tempol treated Akita had attenuated expression of NOX-4 and Trx suggesting amelioration of oxidative stress in Akita. The mitigation of cardiomyopathy by tempol is reinforced by attenuation of MMP-9, TIMP-3 and induction of TIMP-4 (Figure 10). It suggests that MMP-9 and TIMP-3, -4 are largely involved in diabetic cardiomyopathy in Akita. These findings support the anti-oxidant effect of tempol [43]. The clinical trails failed to provide any beneficial effect of anti-oxidant treatment (specifically vitamin C and E) for cardiovascular therapy in Heart Outcomes Prevention Evaluation (HOPE), Study to Evaluate Carotid Ultrasound changes in patients treated with Ramipril and vitamin E (SECURE), and Secondary Prevention with Antioxidant of Cardiovascular disease in End stage renal disease (SPACE) [45]. Nonetheless, these trails suffer from several limitations. One of the limitations was that these trials were not designed specifically for assessing the impact of anti-oxidant on diabetes patients. Notably, in specific trial for oxidative stress, where patients on hemodialysis were exposed to high oxidative stress and supplemented with vitamin E showed remarkable response [64] suggesting that anti-oxidant could ameliorate the pathological condition. Although, plethora of evidences supports the anti-oxidant effect of tempol [65-68], hitherto no clinical data is available for its effect on diabetic cardiomyopathy. Our findings extend support to the fact that PPARγ and tempol are promising therapeutic targets for diabetic cardiomyopathy.

**Figure 11:**
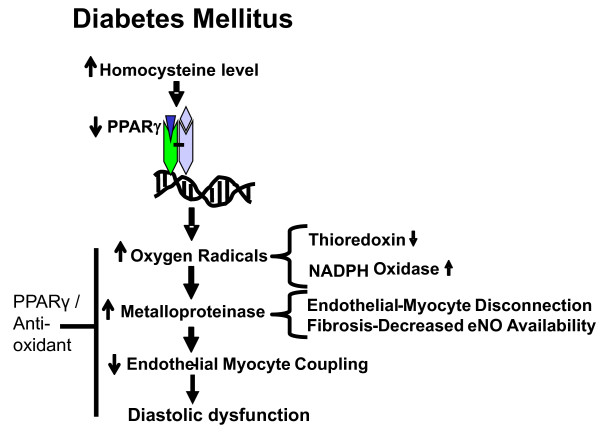
**A model for diabetic cardiomyopathy**. In diabetes, elevated level of homocysteine induces oxidative radicals by antagonizing PPARγ that activates matrix metalloproteinases (MMPs), which in turn causes fibrosis and endothelial-myocytes disconnection. It results into endothelial-myocytes uncoupling that ultimately leads to diastolic dysfunction. PPARγ agonist and anti-oxidants mitigate the effect of oxidative radicals, inhibit the activity of MMPs and thereby ameliorate diastolic dysfunction.

Based on our findings, we conclude that in diabetes, HHcy antagonizes PPARγ and induces production of oxygen radicals that enhances MMP-9 and inhibits TIMP-4, which in turn causes matrix remodeling, E-M uncoupling and fibrosis impairing diastolic functions. There is synergism between HHcy and hyperglycemia in diabetic cardiomyopathy. The treatment with PPARγ agonist (CZ) and anti-oxidant (tempol) mitigates oxidative stress and thereby ameliorates diastolic dysfunction in diabetes (Figure 11).

## Abbreviations

ACH: acetylcholine; CBS: Cystathionine beta synthase; CZ: Ciglitazone; ECM: Extracellular matrix; EDD: End diastolic diameter; E-M: Endothelial- myocyte; Hcy: Homocysteine; HHcy: Hyperhomocysteinemia; HPLC: High- performance liquid chromatography; Ins2: Insulin 2; LV: Left ventricle; LVH: left ventricular hypertrophy; MMP: Matrix metalloproteinase; NOX4: NADPH oxidase 4; PPAR: Peroxisome proliferator- activated receptor; ROS: Reactive oxygen species; SD: standard deviation; TIMP: Tissue inhibitor of metalloproteinase; Trx: Thioredoxin; WT: Wild type.

## Competing interests

The authors declare that they have no competing interests.

## Authors' contributions

SCT conceived the study. SCT and IGJ designed the study and corrected the manuscript. PKM wrote the manuscript. NT, US and PKM contributed in performing the experiments, analyzing the data and interpreting the results. All authors read and approved the final manuscript.
